# Macrophage polarization in Sjögren’s syndrome: from mechanisms to therapeutic translation

**DOI:** 10.3389/fimmu.2026.1819964

**Published:** 2026-08-04

**Authors:** Ke-Ying Zhu, Xi-Meng Li, Qian-Lei Xu, Huan Li, Cheng-Zhi Wang, Shang-Wen Wu, Zhi-Na Zhao, Song-Wei Li, Hui-Jun Guo

**Affiliations:** 1 The First Affiliated Hospital of Henan University of Chinese Medicine, Zhengzhou, China; 2 Henan Provincial Key Laboratory of Traditional Chinese Medicine for Viral Diseases, Zhengzhou, China; 3 The First Clinical Medical School, Henan University of Chinese Medicine, Zhengzhou, China

**Keywords:** clinical translation, M1/M2 imbalance, macrophage polarization, regulatory network, Sjögren’s syndrome, traditional Chinese medicine

## Abstract

Sjögren’s syndrome (SS) is a chronic systemic autoimmune disease characterized by damage to exocrine glands, and no effective treatment is currently available to reverse glandular fibrosis. As core effector cells of innate immunity, macrophages play a pivotal role in the dynamic imbalance of polarization states, which critically influences inflammation initiation, tissue injury, and irreversible fibrosis in SS. This review systematically delineates the heterogeneity of M1/M2 macrophage subsets, their spatiotemporal dynamics, and their functional transitions across different pathological stages of SS: M1 predominance drives early pro-inflammatory damage, M1/M2 interplay sustains chronic inflammation in the middle stage, and aberrant M2 polarization promotes late-stage glandular fibrosis. We further dissect the multi-level regulatory network underlying macrophage polarization imbalance, including immune cell crosstalk, core signaling pathways, non-coding RNA epigenetic regulation, and immunometabolic reprogramming. The in-depth mechanisms by which M2 macrophages drive the progression of glandular fibrosis are also summarized. In this context, we describe how certain traditional Chinese medicine (TCM) formulas and monomeric compounds can effectively restore the balance of macrophage polarization by targeting relevant pathways and molecular nodes. Moreover, we analyze the major bottlenecks currently hindering the clinical translation of macrophage-targeted strategies and propose innovative approaches, including tissue-specific delivery, spatiotemporally tailored modulation, integrated TCM-western medicine therapy, biomarker-guided patient stratification, and early-window intervention. Finally, we discuss the potential of cutting-edge technologies such as single-cell multi-omics, spatial transcriptomics, gene editing, and organoid models to decode the heterogeneity of macrophage subsets and their interactions with the microenvironment. Integration of these multidisciplinary tools will facilitate the development of safe, effective, and personalized therapeutic strategies targeting macrophage polarization for SS.

## 1 Introduction

Sjögren’s syndrome (SS) is classified as primary SS (pSS) and secondary SS. The global incidence of SS is increasing year by year; in China, the prevalence of pSS is approximately 0.45%, making it a common autoimmune connective tissue disease in clinical practice. Secondary SS often occurs in association with other autoimmune diseases such as rheumatoid arthritis and systemic lupus erythematosus. The core symptoms of SS are dry mouth and dry eyes, accompanied by persistent systemic inflammation that may affect multiple organs (lungs, kidneys, blood), and patients have a significantly increased risk of developing lymphoma, which severely impairs quality of life and long-term prognosis (1–4).

The core pathological features of SS include breakdown of immune tolerance, excessive activation of innate immunity, and dysregulation of adaptive immunity, ultimately leading to chronic persistent inflammation and irreversible fibrosis of exocrine glands (5–7). This pathological process is closely linked to aberrant secretion of type I interferon (IFN-I) and pro-inflammatory cytokines such as IL-6, IL-12, and IL-17; high expression of B-cell activating factor (BAFF); sustained activation of key pathways including NF-κB and JAK-STAT; and abnormal formation of ectopic germinal centers in the glands (8–10). Current conventional clinical treatments mainly target single inflammatory mediators; they provide only temporary symptom relief without halting disease progression or glandular fibrosis, and long-term use is associated with notable side effects, limited efficacy, and high relapse rates.

Recent basic and clinical studies have confirmed that macrophages are central hubs regulating immune dysregulation and tissue damage in SS, and the dynamic imbalance of their polarization directly determines inflammation initiation, tissue injury, repair dysregulation, and fibrosis progression (11–14). Existing reviews are mostly based on the traditional static M1/M2 dichotomy and have not fully explained the subset heterogeneity or spatiotemporal plasticity of macrophages; moreover, they lack systematic integration and analysis of multi-level regulatory networks, clinical translation obstacles, deep mechanisms of glandular fibrosis, and the role of traditional Chinese medicine (TCM) in modulating these processes. Therefore, this review provides a systematic overview of dynamic macrophage polarization imbalance, multi-level regulatory networks, fibrosis-associated interactions, clinical translation challenges and innovative strategies, and TCM-based regulation, with the aim of summarizing current knowledge, identifying gaps, and outlining future research directions to support precision immuno-intervention in SS.

## 2 Pathogenesis of Sjögren’s syndrome and the central regulatory role of macrophages

**Figure 1:**
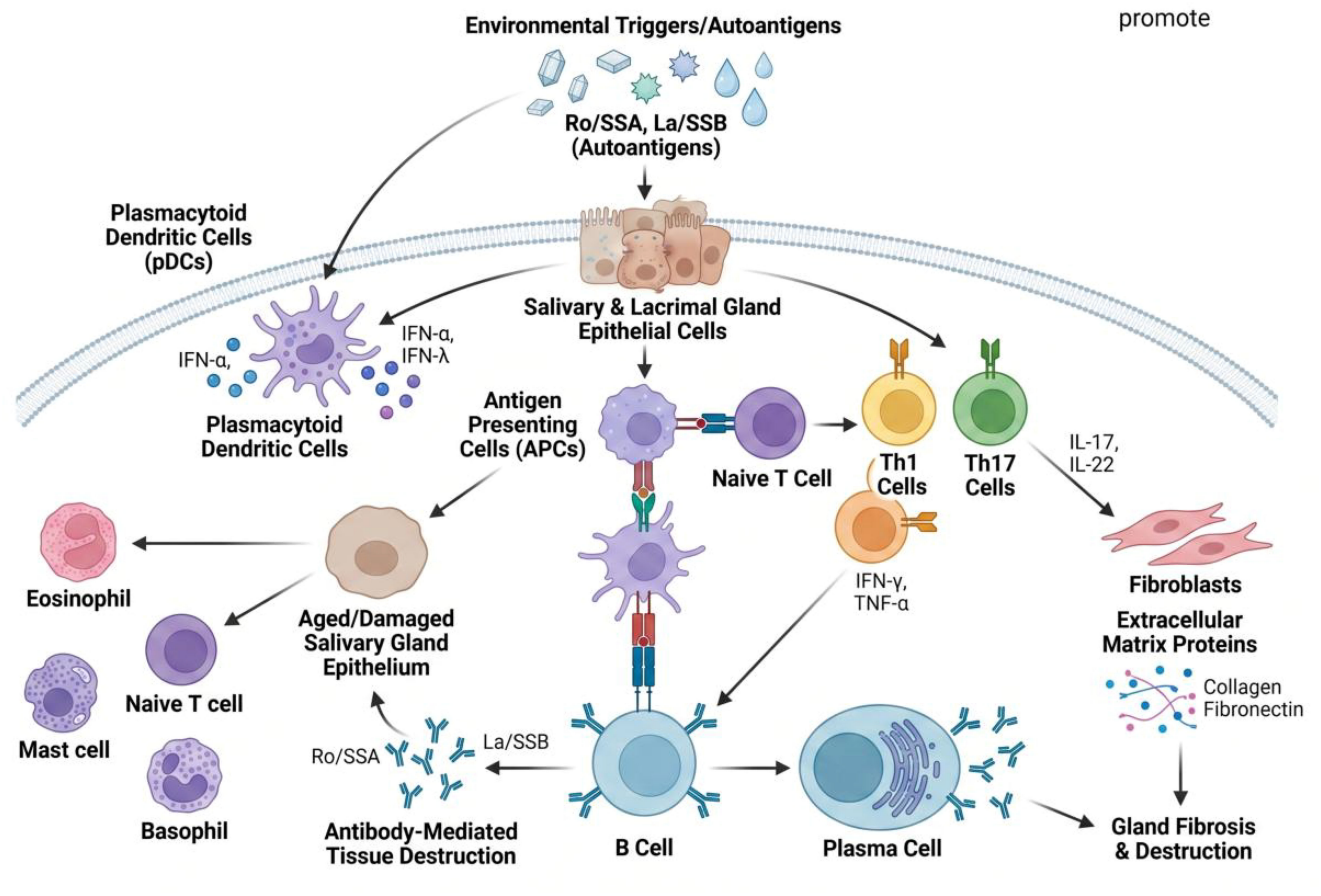
The pathogenesis of Sjögren’s syndrome.

The pathogenesis of SS involves multiple factors, including genetic susceptibility, viral infection, endocrine disorders, and environmental triggers. These stimuli disrupt immune tolerance and expose glandular autoantigens, leading to sustained type I interferon responses and subsequent aberrant activation, proliferation, and infiltration of T and B lymphocytes, ultimately causing structural destruction and functional failure of exocrine glands (15, 16). As key effector and regulatory cells of innate immunity, macrophages participate throughout the entire pathological process of SS and serve as a central hub linking immune dysregulation with tissue injury (**Figure 1**) (14).

**Figure 2:**
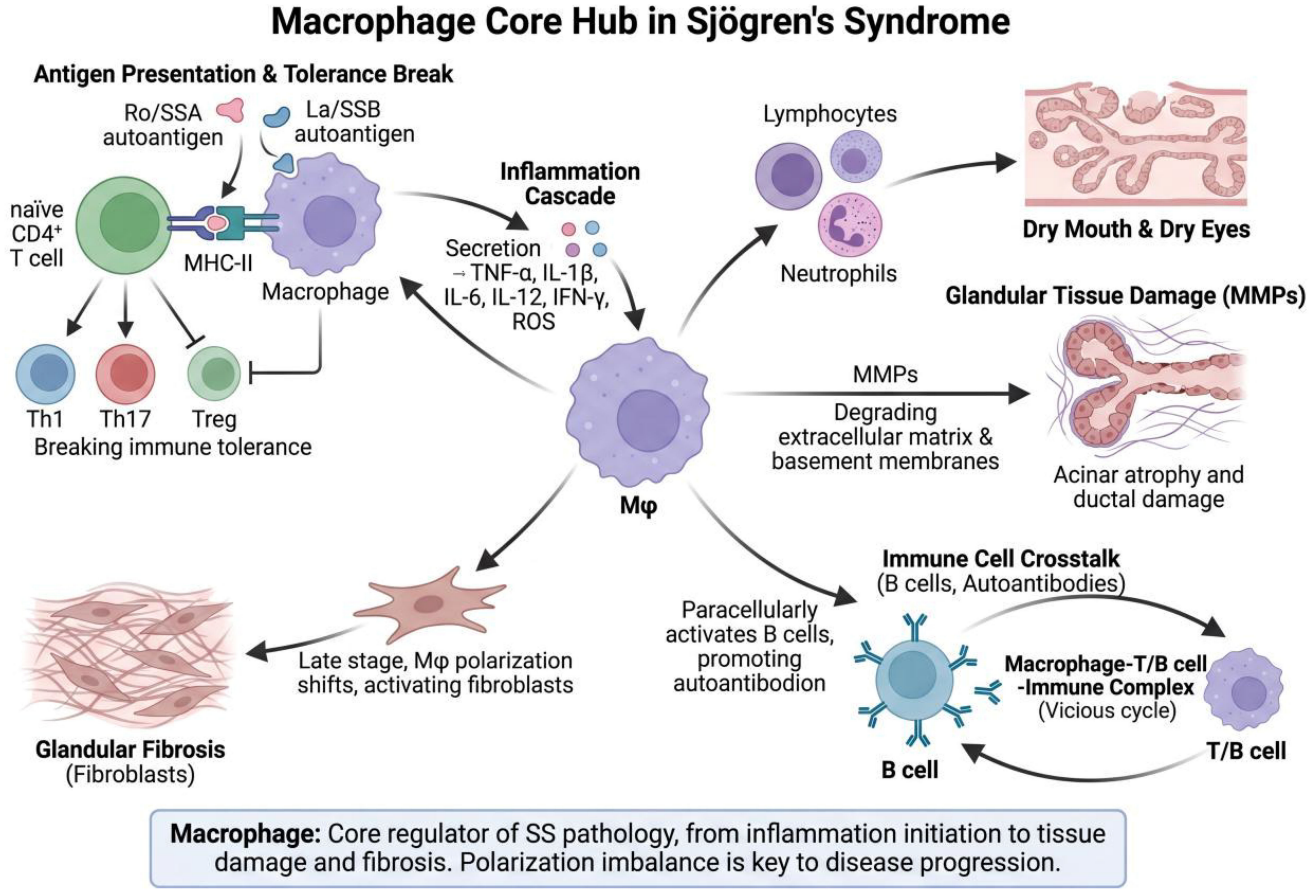
The core regulatory role of macrophages in Sjögren’s syndrome.

Macrophages infiltrating the exocrine glands specifically capture and present characteristic autoantigens (Ro/SSA, La/SSB) via major histocompatibility complex class II (MHC II) molecules to CD4^+^ T cells, inducing abnormal differentiation of Th1 and Th17 cells and suppressing regulatory T-cell (Treg) function, thereby breaking immune tolerance and initiating autoimmune inflammation (17). Activated macrophages secrete large amounts of pro-inflammatory cytokines, including tumor necrosis factor-α (TNF-α), IL-1β, IL-6, IL-12, and IFN-γ, as well as reactive oxygen species (ROS), recruiting additional lymphocytes, neutrophils, and other inflammatory cells into the glandular tissue and creating a positive feedback loop that amplifies local inflammation. In addition, macrophages produce matrix metalloproteinases (MMPs) and other bioactive mediators that degrade the extracellular matrix and basement membrane, leading to acinar atrophy and ductal damage and directly impairing saliva and tear secretion (18, 19). These activated macrophages also influence B-cell proliferation, differentiation, and activation through paracrine effects, promoting autoantibody production and sustaining systemic autoimmunity (19, 20). In the middle and late stages of the disease, the polarization state of macrophages shifts, and through regulation of fibroblast activation, aberrant collagen deposition, and extracellular matrix (ECM) remodeling, they drive the transition from chronic inflammation to irreversible fibrosis, becoming a key driver of long-term organ damage in SS (21). Collectively, macrophages are not merely inflammatory effector cells but central regulators that orchestrate inflammation initiation, amplification, tissue injury, and fibrosis throughout the disease course; their dynamic polarization imbalance is critical for disease persistence, relapse, and refractoriness (**Figure 2**).

## 3 Heterogeneity and dynamic imbalance of macrophage polarization in SS

### 3.1 Core phenotypes and functional characteristics of M1/M2 macrophage polarization

Macrophages possess strong environmental plasticity and subset heterogeneity, allowing them to dynamically adjust their polarization phenotype and function according to local tissue signals. They are classically divided into pro-inflammatory M1 and anti-inflammatory/repair M2 types; M2 cells can be further subdivided into M2a (tissue repair), M2b (immunoregulation), M2c (immunosuppression), and M2d (tumor-associated) subsets, each with distinct phenotypic and functional characteristics. In the SS microenvironment, macrophage polarization is not static but exhibits a dynamic imbalance characterized by M1 predominance in the early stage, M1/M2 co-existence and interplay in the middle stage, and M2-driven pro-fibrotic activity in the late stage.

M1 pro-inflammatory macrophages are primarily activated by signals such as IFN-γ, TNF-α, lipopolysaccharide (LPS), and granulocyte-macrophage colony-stimulating factor (GM-CSF), they specifically express high levels of surface markers and functional proteins such as CD86, CD40, and iNOS, and secrete pro-inflammatory mediators including TNF-α, IL-1β, IL-6, IL-12, and ROS (22–24). Their core functions are to initiate and amplify inflammatory responses, eliminate pathogens, drive Th1 immune responses, and mediate tissue damage, making them key effector cells for inflammation initiation in autoimmune diseases. M2 anti-inflammatory/repair macrophages are primarily induced by IL-4, IL-13, IL-10, TGF-β, and M-CSF; they express CD206, CD163, and Arg-1, and secrete anti-inflammatory/repair factors such as IL-10 and TGF-β (25–27). Their classic functions are to suppress excessive inflammation, promote tissue repair, and maintain immune homeostasis, but in chronic diseases they can aberrantly mediate matrix remodeling and fibrosis (**Table 1**).

**Table 1:** Phenotypes, stimulants, biomarkers, secretions, and functions of macrophages.

Macrophage phenotype	Stimulants	Biomarkers	Secretions	Functions
M1 (classically activated macrophages)	IFN-*γ*, LPS, TNF-*α*, GM-CSF	CD86, CD40, CD38, NF-*κ*B, STAT1	TNF-α, IL-1α, IL-12, IL-23, IL-1β, IL-6, ROS, and RNS	Promote Th1 immune response, promote inflammatory response, fight pathogens, and inhibit the occurrence and development of tumors
M2a (wound healing macrophages)	IL-4, IL-13, M-CSF	CD206, IL-1R, CCL17, Fizz1, STAT6	TGF-*β*, IL-10, insulin-like growth factor (IGF), and fibronectin	Promote tissue repair and remodeling, promote fibrosis, and promote type II immune response by enhancing polyamines, collagen synthesis
M2b (regulatory macrophages)	Immune complex, TLR agonist, IL-1R agonist	IL-10, CCL1, LIGHT, CD86, SPHK1, TNF-*α*, IL-6, ERK, AP-1	Proinflammatory cytokines (IL-1*β*, IL-6, and TNF-*α*), anti-inflammatory cytokine (IL-10 and low levels of IL-12)	Involve in proinflammatory and anti-inflammatory responses, immunomodulation, and Th2 activation
M2c (acquired inactivated macrophages)	Glucocorticoids, TGF-*β*, IL-10	CD163, Mer receptor tyrosine kinase (MerTK), STAT3	IL-10, TGF-*β*	Immune tolerance and tissue repair, suppress inflammation, promote phagocytosis and cholesterol efflux
M2d (tumor-associated macrophages	TLR, adenosine A2A receptor *γ*, IL-6	Vascular endothelial growth factor A (VEGF-A), HIF-1*α*	Proteolytic enzymes (MMP-2), growth factors (VEGF), and anti-inflammatory mediators (TGF-*β*)	Beneficial for angiogenesis and tumor metastasis

### 3.2 M1 macrophage polarization as a key driver of early inflammation in SS

M1 macrophage polarization is a major driver of early inflammation initiation and persistent amplification in SS (28–30). Studies show that M1 macrophages predominate during the early stage of SS, producing pro-inflammatory cytokines such as TNF-α, IL-6, IL-1β, and IL-12, and further activating CD4^+^ T cells to differentiate into the Th1 lineage, leading to submandibular gland inflammation and the establishment/maintenance of a chronic inflammatory microenvironment (13, 31). Moreover, M1 macrophages induce a proteolytic microenvironment by secreting MMPs and plasmin, directly causing tissue destruction, repair dysfunction, and impaired glandular function, thereby sustaining chronic inflammation and causing persistent injury (18). As antigen-presenting cells, they present autoantigens to T lymphocytes, breaking immune tolerance and launching specific immune attacks against glandular tissue, which is a central step in glandular injury (13).

Recent work using single-cell RNA-seq (scRNA-seq) of peripheral blood mononuclear cells (PBMCs) revealed that damaged glandular tissues in pSS contain abundant tissue-resident macrophages, and high abundance of pro-inflammatory M1 macrophages is accompanied by high levels of other infiltrating immune cells, senescence-associated secretory phenotypes, and marked metabolic reprogramming; these M1 cells play a key role in peripheral inflammation by modulating the release of inflammatory cytokines and phagocytosis (13). Lu Zhao et al. demonstrated that reducing M1 macrophage expression alleviates dry eye symptoms in SS (12). Another study found that human umbilical cord mesenchymal stem cells (MSCs) effectively reduce chronic inflammation and improve clinical symptoms in SS by inhibiting the expression of iNOS, TNF-α, and IL-6 in pro-inflammatory M1 macrophages (32). From the perspective of glycolytic reprogramming and epigenetics, it was shown that IFN-γ significantly promotes the expression of methyltransferase-like 3 (METTL3) in macrophages and the N^6^-methyladenosine (m^6^A) modification level of signal transducer and activator of transcription 1 (STAT1); inhibiting METTL3 expression markedly reduces STAT1 m^6^A modification and the proportion of M1-polarized macrophages. Treatment with the TCM formula Meihua Shengjin Yin significantly inhibited M1 macrophage polarization and improved inflammation (33). Early studies analyzing the relationship between serum pro-inflammatory cytokine levels and disease activity found that M1 macrophages are abundant in the early stage of SS, produce TNF-α, IL-6, and IL-12, activate CD4^+^ T cells, promote Th1 differentiation, and aggravate glandular inflammation (34). Moreover, high-mobility group box 1 (HMGB1) was identified as a key factor driving M1 polarization of macrophages in SS salivary glands, leading to glandular dysfunction and injury (35). Using transcriptome sequencing and bioinformatics combined with clinical data, key genes highly expressed in SS patients were found to be associated with follicular helper T cells (Tfh), memory B cells, and M1 macrophages (36). Thus, M1 macrophage polarization plays a predominantly pro-inflammatory role in SS. However, current studies have limitations: most human sample analyses are correlative and lack causal validation, and *in vitro* and mouse models cannot fully replicate the complex human glandular microenvironment, limiting the translation of basic findings to clinical features.

### 3.3 Dual role of M2 polarization in SS progression

M2 macrophages have a significant dual effect during the course of SS and are key cells mediating the transition from acute inflammation to chronic fibrosis, with their functions changing dynamically with disease stage. In the early stage, appropriately activated M2 macrophages exert protective effects by secreting anti-inflammatory factors such as IL-10 and TGF-β, inhibiting excessive M1 activation, reducing glandular inflammation, promoting tissue repair, and maintaining local immune homeostasis, thereby preventing uncontrolled inflammation. In the middle and late stages, persistently aberrant M2 polarization becomes pathogenic and is a core driver of irreversible glandular fibrosis.

A recent Chinese study confirmed that in SS mice, M1 markers (iNOS, CD86) are upregulated while M2 markers (Arg-1, CD206) are downregulated; the drug iguratimod inhibited M1 polarization and promoted M2 polarization via the MAPK pathway, effectively suppressing submandibular gland inflammation, protecting glandular function, and alleviating symptoms (37). Another study demonstrated that the paeoniflorin derivative CP-25 activated the TAM receptor downstream suppressors of cytokine signaling 1 and 3 (SOCS1/3), thereby inhibiting the JAK1-STAT1 pathway and promoting M2 polarization, achieving therapeutic effects in SS (38). MSC-derived small extracellular vesicles (MSC-sEVs) effectively induced an anti-inflammatory M2 phenotype and increased the proportion of Tregs both *in vivo* and *in vitro*, reducing inflammation and ameliorating tissue damage (39). Histological and immunofluorescence analysis of labial salivary gland samples from 22 pSS patients suggested that CXCR3^+^CD163^+^ M2 macrophages may contribute to anti-inflammatory function in pSS lesions (40). Recent cellular experiments showed that leukocyte tyrosine kinase (LTK) deficiency induced M2 polarization of macrophages by targeting CXCL13, thereby improving dry mouth and dry eye symptoms and slowing disease progression (41). M2 macrophages secrete chemokines such as CCL18 and CCL22, specifically recruiting Th2 cells, B lymphocytes, and plasma cells into the glandular tissue, promoting autoantibody production and exacerbating acinar cell injury. Some authors have pointed out that YKL-40 secreted by M2 macrophages induces fibroblast activation, collagen deposition, and glandular fibrosis, further destroying exocrine gland structure and function (42); concurrently, activated fibroblasts secrete IL-6, TNF-α, and other factors that reciprocally promote M2 polarization, forming an “M2 macrophage-fibroblast” positive feedback loop that sustains chronic inflammation and fibrosis. In SS mouse models, macrophages in the salivary glands produce high levels of the B-cell chemokine CXCL13, which may facilitate the formation of ectopic lymphoid tissue centers within the salivary glands (43). A recent study revealed a potential exosome-based therapeutic strategy: exosomes from hypoxic-pretreated bone marrow MSCs (BMSCs) promoted M2-like macrophage polarization via PPARγ, thereby repairing SS-induced skin damage (44). Nevertheless, current research has notable gaps: the spatiotemporal distribution and functional specificity of M2 subsets in SS remain unclear, subset heterogeneity is not fully resolved, dynamic tracking data of human glandular fibrosis are lacking, and the precise mechanisms by which M2 polarization mediates fibrosis require further validation.

### 3.4 Polarization imbalance: from static dichotomy to dynamic network dysregulation

The traditional M1/M2 dichotomy represents a static, simplified biological classification that cannot accurately describe the true pathological state of macrophages in SS. Macrophages in the SS glandular microenvironment exhibit pronounced spatiotemporal dynamics, subset heterogeneity, and functional mixing; absolute M1 or M2 phenotypes do not exist. Early disease is characterized by M1 dominance with core inflammatory injury; in the middle stage, M1 and M2 co-exist, reflecting a dynamic balance between inflammation and repair; in the late stage, M2 dominance prevails, with reduced inflammation but continuously worsening fibrosis. The essence of macrophage polarization imbalance is a plasticity disorder driven by dynamic regulation of cytokines, metabolites, and matrix mechanical signals within the glandular microenvironment. This dynamic dysregulation underlies the chronic, progressive, irreversible nature of SS.

## 4 Multi-level regulatory network of macrophage polarization in SS

Macrophage polarization imbalance in SS is not the result of a single pathway but a complex network involving immune cell interactions, core signaling pathways, non-coding RNA epigenetic regulation, and immunometabolic reprogramming. Each level acts in coordination to drive polarization dysregulation and disease progression.

### 4.1 Immune cell interaction network

Various immune and stromal cells in the glandular microenvironment communicate via secreted cytokines, membrane-bound molecules, and exosomes, forming a complex cellular interaction network that precisely regulates macrophage polarization (35).

Th1/Th17 cells secrete IFN-γ, IL-17, and IL-22, strongly inducing M1 polarization while inhibiting M2 polarization, thereby amplifying glandular inflammation and maintaining the M1-dominant state (45–49).

Treg cells normally secrete IL-10 and TGF-β, promoting M2-type repair polarization and suppressing M1 over-activation to maintain immune homeostasis; in SS, Treg numbers and function are reduced, and this negative regulation is lost, further exacerbating polarization imbalance (50, 51).

B cells secrete autoantibodies, IL-10, and BAFF; through Fc receptor engagement, they activate M1 pro-inflammatory phenotypes and also induce M2 fibrotic phenotypes, participating in both immune dysregulation and fibrosis. In pSS, high expression of IL-18 by macrophages is observed in B-cell-rich areas and germinal center-like structures (52). CCR1^hi^/CCL5^hi^ macrophages in SS salivary glands promote T- and B-cell infiltration and activation; *in vitro* studies indicate that macrophages cooperate with T cells and enhance B-cell recruitment via CCL5, playing an important role in SS immunopathology (14, 53).

Dendritic cells (DCs) present autoantigens to naïve T cells, inducing their differentiation toward pro-inflammatory phenotypes; DCs also secrete IFN-γ and IL-12, which synergistically enhance M1 polarization and function (54). Local production of type I IFN by DCs induces macrophages to produce CXCL13, which then promotes accumulation of CXCR5^+^CD19^+^ B cells, aggravating disease (55).

Glandular fibroblasts secrete TGF-β and IL-6, specifically promoting M2 fibrotic polarization and forming a positive feedback loop that accelerates irreversible glandular damage.

### 4.2 Regulation by core cytokines and signaling pathways

#### 4.2.1 Core pathways regulating M1 polarization

JAK-STAT1 pathway: IFN-γ binding to its receptor activates JAK1/2 kinases, leading to STAT1 phosphorylation and nuclear translocation, which specifically transcribes M1 target genes such as iNOS, TNF-α, and IL-6 (56, 57).

NF-κB pathway: Activated by LPS, autoantigens, and DAMPs through TLR4/MyD88 signaling, the IKK complex is activated, leading to IκB degradation and NF-κB nuclear translocation, which transcribes a large array of pro-inflammatory cytokines, driving M1 polarization and inflammation amplification (58, 59).

MAPK pathway: Aberrant activation of p38, JNK, and ERK kinases cooperates with NF-κB and STAT1 pathways to amplify M1 signals, enhance pro-inflammatory gene expression, and accelerate disease progression (58, 59).

#### 4.2.2 Core pathways regulating M2 polarization

JAK-STAT6 pathway: IL-4/IL-13 binding activates JAK1, leading to STAT6 phosphorylation and nuclear translocation, which transcribes M2 genes such as CD206, Arg-1, and TGF-β (60, 61).

STAT3 pathway: IL-10 activates STAT3, promoting M2 polarization while inhibiting M1 responses, mediating immunosuppression and tissue repair (33).

Akt/FoxO1 pathway: TGF-β activates Akt, leading to FoxO1 phosphorylation and inactivation, which promotes M2 polarization and activates fibrosis-related genes, participating in glandular repair and fibrosis (62, 63).

PPARγ pathway: IL-4 induces PPARγ expression, which cooperates with STAT6 to stabilize the M2 phenotype and mediate tissue repair and matrix remodeling (64).

### 4.3 Epigenetic regulation by non-coding RNAs

LncRNAs and miRNAs fine-tune the expression of core pathway genes and targets at the post-transcriptional level, serving as important epigenetic regulators of polarization balance; their abnormal expression directly induces polarization imbalance.

#### 4.3.1 Non-coding RNAs regulating M1 polarization

Wang et al. found that TMEVPG1 is highly expressed in SS patients and correlates with anti-SSA antibodies, erythrocyte sedimentation rate, and IgG (65). TMEVPG1 is located on the opposite DNA strand of the IFN-γ gene (66), and IFN-γ activates the JAK-STAT pathway to promote M1 polarization. Inamo et al. reported that LINC00487 is upregulated in B-cell subsets of SS patients, and its upstream regulator is IFN-α. Ye et al. observed increased NEAT1 expression in peripheral T lymphocytes of SS patients; knockdown of NEAT1 suppressed TNF-α and CXCL8 expression, and TNF-α itself promotes M1 polarization (67). Peng et al. analyzed 1,772 lncRNAs and found that NRIR, BISPR, LINC00426, and CYTOR were upregulated while TPTEP1 was downregulated in SS patients, and these lncRNAs are involved in multiple pathways linked to SS pathogenesis, including NF-κB, MAPK, and JAK-STAT signaling (68).

Several miRNAs also regulate M1 polarization. miR-146a expression is increased in salivary glands and PBMCs of SS patients and mouse models (69). As an NF-κB-dependent miRNA, miR-146a reduces NF-κB-related proteins, and inhibition of miR-146a promotes M1 polarization. Reduced miR-31-5p levels are associated with M1 macrophage activation, and abnormal expression of m^6^A regulators is closely linked to SS and other diseases (70). Upregulation of m^6^A-modified miR-31-5p inhibits the p38 MAPK pathway, downregulates pro-inflammatory factors (NOS2, IL-1β, TNF-α) and upregulates Arg-1, CD206, and IL-10, thereby suppressing M1 and promoting M2 polarization, restoring the M1/M2 balance, reducing lymphocyte infiltration, alleviating local damage, and improving dry eye symptoms (12). Thus, non-coding RNAs can activate NF-κB and p38 MAPK pathways, directly or indirectly, to induce M1 polarization, leading to excessive release of inflammatory cytokines and exacerbation of SS symptoms.

#### 4.3.2 Non-coding RNAs regulating M2 polarization

Differential expression of lncRNAs and miRNAs in pSS is involved in disease pathogenesis (69, 71). In a rabbit model of autoimmune dacryoadenitis, small extracellular vesicles derived from human umbilical cord MSCs promoted M2 polarization and induced Tregs via miR-100-5p, alleviating autoimmune dacryoadenitis (37). Inhibition of miR-125b in aging extracellular vesicles reduced CD38 expression, increased CD206 expression, and lowered the CD38/CD206 ratio, thereby promoting M2 polarization; this mechanism also downregulated IL-17A and IL-21, suppressed Th17 differentiation, and improved glandular function in SS patients (72). Overexpression of miR-100-5p decreased NOS2 and interferon regulatory factor 5 (IRF5) expression and increased CD206, Arg-1, and Krüppel-like factor 4 (KLF4) expression, driving M2 polarization and alleviating inflammation, dry eye severity, and conjunctival symptoms (37).

### 4.4 Immunometabolic reprogramming

Macrophage polarization is tightly coupled with cellular metabolic patterns. M1 pro-inflammatory macrophages rely mainly on glycolysis to rapidly generate ATP and ROS, providing energy and substrates for pro-inflammatory functions. M2 repair/fibrotic macrophages primarily utilize oxidative phosphorylation and fatty acid oxidation, which support tissue repair and matrix remodeling. In the SS glandular microenvironment, disturbances in glucose, lipid, and amino acid metabolism drive aberrant metabolic reprogramming via core metabolic signaling molecules such as HIF-1α, AMPK, and mTOR, leading to persistent M1 over-activation and aberrant activation of M2 fibrotic phenotypes, establishing a vicious cycle that aggravates inflammation and fibrosis.

### 4.5 Integrated analysis of the multi-level regulatory network

**Figure 3:**
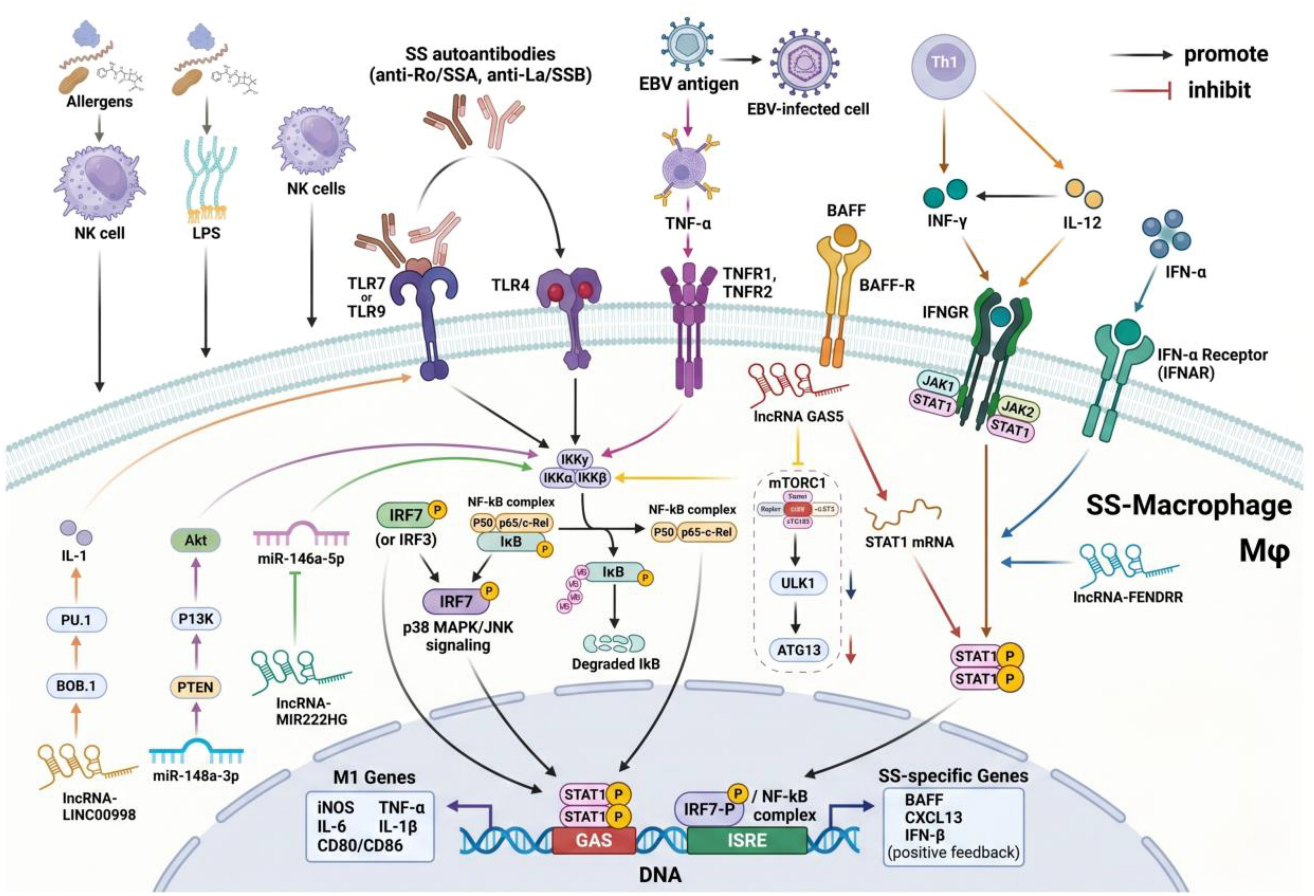
Mechanism of M1 polarization in Sjogren’s syndrome.

**Figure 4:**
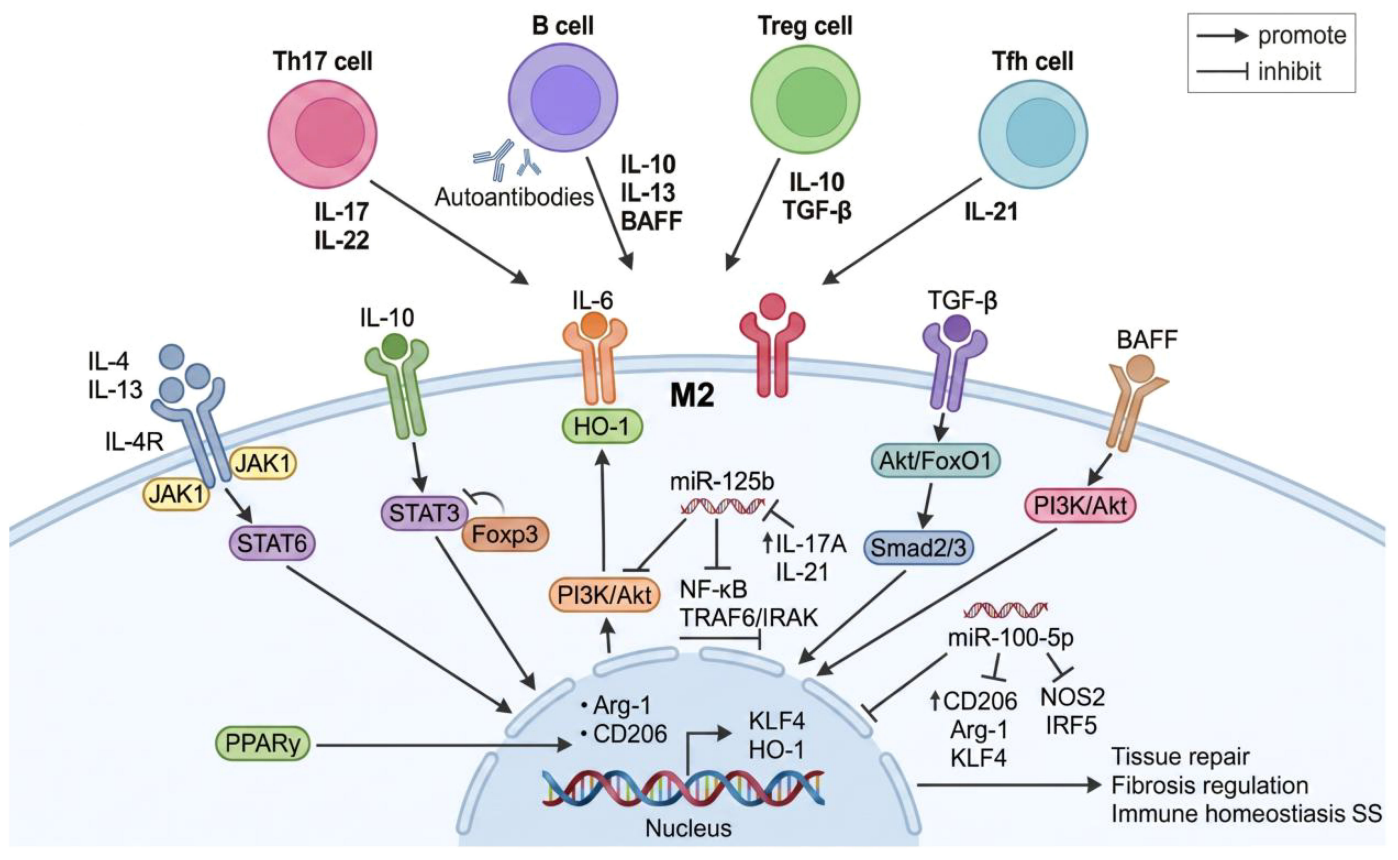
Mechanism of M2 polarization in Sjögren syndrome.

These regulatory mechanisms do not act independently but are interconnected, forming a complete dynamic network: autoantigens and DAMPs trigger the TLR/NF-κB pathway, inducing M1 polarization and pro-inflammatory cytokine release; IFN-γ from M1 cells further activates the JAK-STAT1 pathway, amplifying pro-inflammatory signals; anti-inflammatory signals such as IL-4 and IL-10 activate STAT6/STAT3 pathways, initiating M2 polarization; non-coding RNAs fine-tune core pathway activity; immunometabolic reprogramming supplies energy and substrates; and various immune and stromal cells modulate polarization signals through cellular crosstalk. The spatiotemporal dysregulation of this multi-level network is the fundamental cause of macrophage polarization imbalance and disease progression in SS (**Figures 3**, **4**).

## 5 Deep mechanisms of macrophage interaction with glandular fibrosis in SS

Irreversible glandular fibrosis is the core pathological change leading to disability and organ failure in late-stage SS, and it is a major challenge for clinical management. Macrophages are key hub cells linking chronic inflammation and fibrosis. In particular, aberrantly activated M2 macrophages drive all stages of fibrosis through multiple mechanisms. Abnormally polarized M2 macrophages secrete large amounts of pro-fibrotic factors (TGF-β1, YKL-40), inducing the transformation of resident fibroblasts into myofibroblasts, which markedly upregulate the synthesis of matrix proteins such as collagen I and III, leading to extensive ECM deposition. Activated fibroblasts reciprocally secrete TGF-β and IL-6, further driving macrophages toward the pro-fibrotic M2 phenotype, forming a vicious cycle that sustains fibrosis progression (73, 74). Second, TGF-β secreted by M2 macrophages induces epithelial-mesenchymal transition (EMT) in glandular epithelial cells, converting secretory epithelial cells into fibroblast-like mesenchymal cells; this results in loss of functional glandular epithelial cells and increases the number of matrix cells, thereby exacerbating fibrotic damage (75, 76). M2 macrophages also regulate aberrant ECM remodeling by differentially secreting MMPs and tissue inhibitors of metalloproteinases (TIMPs), disrupting the balance between matrix synthesis and degradation, inhibiting matrix degradation, and promoting collagen accumulation, ultimately leading to glandular architectural disorganization and sclerotic fibrosis (77, 78). Furthermore, M2 macrophages secrete vascular endothelial growth factor (VEGF), promoting abnormal angiogenesis in the glands, which supplies nutrients for fibrotic tissue proliferation and matrix deposition, thereby accelerating fibrosis (79).

Significant knowledge gaps remain in this field. Most studies have focused on the classical TGF-β pathway; the heterogeneity of fibroblast subsets, direct contact-dependent interactions between macrophages and fibroblasts, and the reverse regulation of macrophage polarization by ECM mechanical signals are poorly understood. Single-cell and spatial omics data from fibrotic human SS glands are lacking, limiting our ability to precisely characterize the spatiotemporal features of the fibrotic microenvironment and hindering the development of targeted anti-fibrotic therapies.

## 6 Mechanisms and advantages of traditional Chinese medicine in regulating macrophage polarization

TCM has a long history in treating SS, following the principles of “holistic treatment and syndrome differentiation”. It offers unique advantages, including multi-target, multi-pathway action, low toxicity, and holistic regulation, which are well-suited to the multi-mechanism dysregulation and chronic progression of SS. Recent basic research has confirmed that various TCM monomers, active ingredients, and formulas can target macrophage polarization balance to exert anti-inflammatory, antioxidant, and gland-repairing effects. Despite these promising advantages, most studies are limited to *in vitro* cell experiments and animal models, lacking validation in large clinical cohorts or randomized controlled trials (RCTs). The core targets, spatiotemporal regulatory mechanisms, and material bases of most TCM active ingredients remain unclear (**Table 2**). Future studies should integrate single-cell omics, spatial transcriptomics, and molecular biology to deeply dissect the molecular mechanisms by which TCM regulates macrophage polarization, promoting clinical translation and standardized application of TCM-based therapies.

**Table 2:** Mechanisms of traditional Chinese medicine formulas, monomers, and active components in regulating macrophages.

TCM formula/monomer	Core effect	Regulated signaling pathway	Specific mechanisms and molecular targets
Meihua Shengjin Yin (33)	Inhibits M1 macrophage polarization	METTL3/STAT1 m^6^A methylation axis	The mechanism is closely related to regulating METTL3-mediated m^6^A methylation modification of STAT1, thereby reducing the secretion of inflammatory cytokines IL-6, IL-23, and TNF-α.
Sha-Shen-Mai-Dong Decoction (80)	Regulates macrophage polarization	Not discussed	Not discussed
Ophiopogon japonicus polysaccharide (OJP) (81)	Inhibits M1 macrophage polarization	TNF-α/NF-κB/NLRP3 signaling axis	The mechanism involves inhibition of the relevant signaling pathway and NLRP3 inflammasome activation, reducing oxidative stress and macrophage apoptosis, thereby alleviating macrophage damage and the secretion of inflammatory factors.
Total glucosides of paeony (TGP) (82)	Inhibits macrophage inflammatory response	TLR4/MyD88/NF-κB signaling pathway	The mechanism lies in regulating the overactivation of the TLR4/MyD88/NF-κB signaling pathway, thereby suppressing the macrophage-mediated inflammatory cascade.
CP-25 (paeoniflorin derivative) (38)	Promotes M2 macrophage polarization	Gas6-TAM-SOCS1/3 axis → JAK1-STAT1 pathway	The mechanism involves activating the TAM receptor family, enhancing SOCS1/3-mediated inhibition of JAK kinases, and suppressing JAK1-STAT1 pathway activation, thus promoting macrophage polarization toward the M2 phenotype.

## 7 The modulatory impact of clinical first-line and second-line therapeutic agents on macrophage polarization equilibrium

In accordance with the domestic diagnostic and treatment guidelines for primary Sjögren’s syndrome and the EULAR recommendations for its management, clinical therapeutic drugs are classified into two principal categories: local symptomatic agents and systemic immunomodulatory drugs. Systemic immunotherapy drugs can be further stratified based on their clinical application hierarchy and mechanisms of action into first-line basic maintenance drugs (e.g., hydroxychloroquine), conventional second-line immunosuppressants, targeted biologics, and JAK small-molecule kinase inhibitors. Glucocorticoids are administered solely for short-term therapy in cases involving severe organ damage and intense systemic inflammation, serving as adjuvant anti-inflammatory agents during the acute phase and excluded from the classification of long-term maintenance medications (83, 84). Various drugs target pivotal signaling pathways implicated in macrophage polarization, including TLR, NF-κB, MAPK, JAK-STAT, PI3K/Akt, and PPARγ, to inhibit M1 pro-inflammatory polarization upstream, induce a protective M2 phenotype, or impede the pathological M2-mediated fibrosis process, thereby restoring the local M1/M2 equilibrium in exocrine glands and offering targeted intervention strategies for immune dysfunction and glandular damage in Sjögren’s syndrome (**Table 3**).

**Table 3:** Regulatory effects of first-line/second-line clinical therapeutic agents on macrophage polarization equilibrium.

Drug category	Representative agents	Clinical indication	Key mechanism	Effect on macrophage polarization	Key effects and clinical implications
First-line baseline therapy	Hydroxychloroquine (35, 83–87)	Long-term baseline maintenance for mild-to-moderate pSS	Inhibits TLR7/9 downstream NF-κB and p38 MAPK pathways; upregulates PPARγ	Predominantly suppresses M1 pro-inflammatory polarization, with mild and weak induction of M2 anti-inflammatory phenotype	1. Molecular level: Downregulates M1 markers (iNOS, CD86), reduces pro-inflammatory cytokines (TNF-α, IL-1β, IL-6, IFN-I), and inhibits HMGB1 release. 2. Repair effect: Only at low doses mildly upregulates M2 markers (Arg-1, CD206) and anti-inflammatory cytokine IL-10.
Second-line conventional immunosuppressants	Iguratimod (88, 89)	Patients with moderate-to-severe active SS who respond inadequately to hydroxychloroquine monotherapy	Inhibits MAPK-STAT1 pathway; upregulates SOCS1/3 and PPARγ	Bidirectional balancing regulation: significantly suppresses M1 overactivation while promoting M2 anti-inflammatory differentiation	1. Animal model evidence: Reduces iNOS expression in submandibular glands and decreases M1 macrophage proportion in spleen. 2. Tissue benefits: Alleviates glandular swelling and lymphocytic infiltration, restores salivary secretory function, and delays glandular fibrosis progression.
	Methotrexate (90)	pSS complicated by polyarthritis	Interferes with cellular folate metabolism; downregulates NF-κB signaling	Selectively inhibits M1 macrophage overactivation, with no significant regulatory effect on M2 polarization	Cell-specific effect: Intracellular accumulation of MTX-PG in M1 macrophages is fourfold higher than in M2, enabling differential regulation of folate metabolism, chemokine, and energy metabolism-related genes.
	Leflunomide (91)	SS with persistent polyarthritis	Scavenges ROS; upregulates PPARγ expression	Primarily promotes M2 anti-inflammatory differentiation	Molecular effects: Upregulates M2 markers (Arg-1, CD206 at both mRNA and protein levels) and IL-10, while downregulating pro-inflammatory mediators (TNF-α, IL-6).
	Mycophenolate mofetil (MMF) (92, 93)	Severe SS with interstitial lung disease, renal involvement, or advanced glandular fibrosis	Competitively inhibits the de novo purine synthesis pathway	Dual regulation: suppresses M1 inflammatory activation and shifts macrophages toward an M2-like reparative phenotype	1. Anti-inflammatory: Reduces pro-inflammatory mediators (IL-1β, TNF-α) in animal models. 2. Repair modulation: Upregulates M2 markers (CD163, CD200R) and blocks monocyte–endothelial cell adhesion. 3. Anti-fibrotic mechanism: Balances M2 secretion of TGF-β and YKL-40, interrupting the M2–fibroblast pro-fibrotic crosstalk.
Glucocorticoids (short-term second-line)	Prednisone, etc (94–96)	Short-term pulse therapy for acute severe inflammatory flares; long-term maintenance is contraindicated	Dose-dependent pathway modulation: high-dose inhibits NF-κB and MAPK; low-dose selectively induces M2c regulatory macrophages	Concentration-dependent bidirectional regulation: high-dose potently suppresses M1; low-dose selectively induces M2c regulatory subset	1. Acute-phase benefit: High-dose rapidly curbs M1-mediated systemic hyperinflammation and controls acute organ damage. 2. Local immune tolerance: Low-dose induces CD163 MerTK M2c cells to secrete IL-10 and TGF-β, maintaining local immune homeostasis in glandular tissues.
Targeted biologics (second-line)	Belimumab, Telitacicept, Rituximab (97–99)	Refractory pSS with persistent B-cell activation, BAFF pathway overactivation, and high autoantibody levels	Neutralizes BAFF; depletes aberrantly activated B cells; remodels the lesional microenvironment	Indirectly bidirectionally modulates macrophage polarization via B-cell–macrophage crosstalk	Restores macrophage polarization balance through regulation of BAFF and autoantibodies; suitable for refractory patients with high peripheral autoantibody levels and BAFF pathway activation.
JAK small-molecule inhibitors (second-line)	Baricitinib, Firditinib (100–103)	Refractory pSS with severe dry eye, progressive interstitial lung disease, or high type I interferon signature	Blocks JAK1/TYK2-STAT1 pro-inflammatory axis; preserves STAT3/STAT6 repair signaling	Precision bidirectional regulation: suppresses IFN-driven M1 polarization while concurrently inducing reparative M2 macrophages	1. Anti-M1 mechanism: Blocks the JAK1/TYK2-STAT1 axis, antagonizes IFN-α/IFN-γ-induced M1 differentiation, and downregulates pro-inflammatory molecules (iNOS, TNF-α, IL-6). 2. Pro-repair mechanism: Preserves intact STAT3/STAT6 signaling, upregulates Arg-1, CD206, and IL-10, and stably induces tissue-reparative M2 polarization.

### 7.1 Primary first-line therapeutic agents

Hydroxychloroquine is endorsed as a foundational first-line therapeutic agent for Sjögren’s syndrome in both national and international clinical guidelines. It is particularly indicated for patients with mild to moderate disease severity, primarily presenting with symptoms of xerostomia (dry mouth), xerophthalmia (dry eyes), fatigue, and mild arthralgia, without visceral involvement (83). Mechanistically, hydroxychloroquine elevates lysosomal pH to obstruct TLR7/TLR9 signaling pathways, thereby suppressing the downstream activation of NF-κB and p38 MAPK cascades (85). It also downregulates the expression of M1 macrophage markers, including iNOS and CD86, and diminishes the release of pro-inflammatory cytokines such as TNF-α, IL-1β, IL-6, and type I interferon (86). Concurrently, it inhibits the extracellular secretion of high mobility group box 1 protein (HMGB1), thereby interrupting a pivotal stimulus for the persistent activation of M1 macrophages within glandular tissues (35). Furthermore, prolonged low-dose administration of hydroxychloroquine can modestly upregulate PPARγ expression and moderately elevate the levels of Arg-1, CD206, and IL-10, fostering a mild anti-inflammatory and reparative microenvironment. Nevertheless, its monotherapy demonstrates limited efficacy in reversing advanced glandular fibrosis (87).

### 7.2 Second-line conventional chemical immunosuppressive agents

For moderate to severe patients who exhibit inadequate response to hydroxychloroquine monotherapy, present with concurrent polyarticular inflammation, or demonstrate mild visceral involvement, second-line immunosuppressive agents such as iguratimod, methotrexate, leflunomide, and mycophenolate mofetil are frequently selected in clinical practice.

The regulatory impact of elamod on macrophage polarization has been substantiated through animal experimentation. Guo Junqiao et al., utilizing the NOD mouse model, discovered that oral administration of elamod markedly diminished the levels of the M1 macrophage marker iNOS in the submandibular glands of SS model mice, as well as the proportion of M1 macrophages within the spleen. This intervention effectively ameliorated submandibular gland enlargement, lymphocyte infiltration, and salivary secretion dysfunction, thereby confirming elamod’s therapeutic potential for Sjögren’s syndrome via the inhibition of M1 macrophage polarization (88). The underlying molecular mechanism encompasses the blockade of the MAPK signaling pathway to suppress STAT1 phosphorylation and reduce the secretion of M1-associated pro-inflammatory cytokines. Concurrently, it upregulates the expression of SOCS1/SOCS3 negative feedback regulators, activates the PPARγ signaling cascade, and promotes the expression of Arg-1 and CD206, thereby rectifying the M1/M2 imbalance in a bidirectional manner and mitigating glandular lymphocyte infiltration and fibrotic alterations (89).

The central mechanism of methotrexate and leflunomide lies in inhibiting the excessive activation of M1 macrophages. As an antifolate agent, methotrexate’s biological activity is contingent upon its conversion into methotrexate polyglutamate (MTX-PG) through FPGS-catalyzed reactions, followed by subsequent intracellular accumulation. Research has demonstrated that the accumulation level of MTX-PG in M1 macrophages is fourfold higher than that in M2 macrophages. MTX primarily induces differential gene expression in M1 macrophages, encompassing genes associated with the folate metabolism pathway, chemokines/cytokines, and energy metabolism, thereby suppressing the activation of the NF-κB pathway by obstructing macrophage folate metabolism (90).

Leflunomide modulates the anti-inflammatory differentiation of macrophages by curbing the generation of reactive oxygen species (ROS) and activating peroxisome proliferator-activated receptor-γ (PPAR-γ). It elevates the mRNA and protein expression levels of anti-inflammatory cytokines, namely IL-10, Arg-1, and CD206, while concurrently and markedly diminishing those of pro-inflammatory cytokines, including TNF-α and IL-6. This observation underscores the significant enhancement of macrophage M2 polarization and anti-inflammatory response by leflunomide (91). Both leflunomide and methotrexate can substantially reduce the local levels of pro-inflammatory factors within glandular tissue; however, methotrexate exhibits a comparatively restricted influence on folate metabolism and gene splicing in M2 macrophages. In contrast, leflunomide predominantly focuses on anti-inflammatory differentiation, with a dearth of compelling evidence supporting its direct induction of M2 polarization. Prolonged monotherapy with leflunomide may impair Treg cell functionality, and thus, in clinical settings, it is frequently administered in conjunction with total glucosides of paeony to offset this limitation.

Mycophenolate mofetil (MMF) is predominantly utilized in the management of critically ill patients presenting with interstitial lung disease, renal injury, and severe glandular fibrosis. MMF exerts its immunosuppressive function by inhibiting purine synthesis. Research has substantiated that MMF can markedly decrease the protein and mRNA levels of pro-inflammatory cytokines, including IL-1β and TNF-α, in the lungs of mice subjected to bleomycin induction, thereby mitigating pulmonary inflammation and the extent of fibrosis (92). Furthermore, a systematic literature review has indicated that MMF is capable of reducing the levels of pro-inflammatory mediators, fostering the M2-like macrophage phenotype, augmenting the expression of M2-like surface markers on macrophages under M1 stimulation, and diminishing the interaction between monocytes and endothelial cells (93).

### 7.3 Glucocorticosteroids

Glucocorticoids predominantly serve as short-term, second-line therapeutic agents during severe acute episodes, with their modulation of macrophage polarization displaying pronounced concentration dependency. High-dose bolus therapy effectively inhibits the NF-κB and MAPK inflammatory signaling pathways, thereby swiftly attenuating intense systemic inflammation mediated by M1 macrophages (94). Conversely, low-dose, prolonged maintenance therapy promotes the generation of CD163^+^MerTK^+^ regulatory M2c macrophages, which secrete IL-10 and TGF-β to sustain local immune tolerance (95). Nevertheless, chronic, high-dose administration excessively stimulates M2 macrophages to mediate collagen deposition, thereby aggravating fibrotic injury to exocrine glands (96). Consequently, glucocorticoids are clinically indicated solely for short-term management of acute organ injury and severe systemic inflammation, and are not advocated for standalone, long-term maintenance therapy.

### 7.4 Targeted biological agents

B-cell-targeted biological agents, including Belimumab, Telitacicept, and Rituximab, constitute second-line therapeutic options for refractory Sjögren’s syndrome (97), indirectly modulating the polarization equilibrium of macrophages through the regulation of upstream microenvironmental factors such as BAFF and autoantibodies (98, 99).

### 7.5 JAK small-molecule inhibitors

JAK inhibitors, including baricitinib and fedratinib, constitute a novel category of second-line targeted therapeutics that directly act upon the pivotal JAK-STAT pathway governing macrophage polarization, thereby facilitating bidirectional modulation (100, 101). These inhibitors obstruct the JAK1/TYK2-STAT1 signaling cascade, counteracting the M1 differentiation instigated by IFN-α and IFN-γ, and subsequently downregulating pro-inflammatory mediators such as iNOS, TNF-α, and IL-6. Concurrently, they preserve the anti-inflammatory signaling mediated by STAT3 and STAT6, upregulate the expression of Arg-1, CD206, and IL-10, and foster the generation of reparative M2 macrophages. This dual mechanism effectively harmonizes the suppression of inflammation with the restoration of glandular tissue, exhibiting commendable polarization regulation and clinical amelioration in cases of refractory Sjögren’s syndrome complicated by severe dry eye and interstitial lung disease (102, 103).

### 7.6 Clinical significance of targeted modulation of macrophage polarization in the stratified management of Sjögren’s syndrome

In conclusion, first-line fundamental immunomodulators can delicately and bidirectionally modulate macrophage polarization and sustain systemic immune homeostasis over an extended period, thus constituting the core therapeutic agents for the long-term management of mild to moderate primary Sjögren’s syndrome. Second-line immunomodulatory agents and targeted pharmaceuticals demonstrate more potent immunosuppressive effects, specifically addressing the two subtypes of polarization imbalance: acute inflammation mediated by overactivated M1 macrophages and glandular fibrosis induced by functionally aberrant M2 macrophages. Based on the macrophage polarization phenotype, a stratified therapeutic approach for primary Sjögren’s syndrome can be formulated: for patients exhibiting substantial infiltration of tissue M1 macrophages and a marked type I interferon response, the addition of JAK inhibitors to hydroxychloroquine can augment anti-inflammatory effects; for those with pronounced glandular fibrosis and a significant presence of pro-fibrotic pathological M2 macrophages, a combination of elamotet and mycophenolate mofetil can be employed to obstruct chronic inflammatory stimuli and decelerate the progression of glandular fibrosis; for refractory patients with notably elevated peripheral autoantibody titers and hyperactivation of the BAFF pathway, owing to the synergistic pro-inflammatory interplay between macrophages and B cells, BAFF-targeted biologics can be utilized to suppress the overall aberrant immune cascade. Presently, notable disparities exist among various drugs in terms of their action targets and the intensity of immunosuppressive effects when regulating the M1/M2 balance, and there remains a paucity of large-scale, prospective, stratified medication cohort studies utilizing the ratio of M1/M2 markers in labial glands and peripheral blood as stratification biomarkers. In the future, research endeavors could concentrate on the synergistic modulation of macrophage polarization by integrating active constituents of traditional Chinese medicine with Western pharmaceuticals, with the objective of devising a comprehensive intervention strategy that amalgamates anti-inflammatory, glandular repair, and anti-fibrotic effects, while concurrently diminishing the dosage of chemical immunosuppressants and mitigating long-term medication-related adverse reactions.

## 8 Clinical translation bottlenecks and novel precision strategies

Targeting macrophage polarization has become an important direction for precision therapy in SS; however, its clinical translation faces multiple challenges. Current small-molecule drugs, biologics, and RNA-based therapies have shown potential in modulating macrophage polarization. For example, JAK inhibitors reduce M1 pro-inflammatory polarization and moderately promote M2 repair polarization via JAK-STAT inhibition (101, 104), but long-term use carries risks of systemic immunosuppression and infection. NF-κB inhibitors block M1 polarization but suffer from off-target effects and hepatorenal toxicity (105). PPARγ agonists stabilize the M2 phenotype but can cause cardiovascular adverse effects (106). In biologics and cell therapies, anti-BAFF antibodies indirectly inhibit M1 polarization but show limited efficacy and high cost (107); MSCs and their exosomes effectively induce M2 polarization and Treg activation (108), but problems such as low delivery efficiency, batch-to-batch variation, and poor stability limit their application. RNA-based therapies can precisely regulate polarization balance (109), but are hindered by immature delivery systems, off-target effects, and RNA instability, and have not yet entered clinical use.

To address these challenges, current clinical strategies should focus on: (1) developing gland-targeted nanocarriers or local injection formulations for precise lesion delivery; (2) implementing spatiotemporally tailored modulation according to disease stage – early inhibition of M1 polarization, mid-stage balancing of M1/M2 homeostasis, and late-stage blockade of M2 pro-fibrotic polarization; (3) integrating TCM with western medicine – rapid anti-inflammation with western drugs and holistic polarization/fibrosis regulation with TCM, to achieve complementary advantages and reduced toxicity (110); (4) focusing on the early inflammatory window to reverse polarization imbalance before fibrosis becomes irreversible; and (5) combining polarization-modulating drugs with B-cell-depleting agents or anti-fibrotic drugs for multi-target synergy to lower monotherapy toxicity and resistance.

Recent advances in single-cell RNA-seq and spatial transcriptomics have overcome the limitations of bulk sequencing, enabling the analysis of macrophage subset heterogeneity and spatial distribution. Single-cell sequencing has revealed that the damaged glands of SS patients contain classical pro-inflammatory M1, repair-oriented M2, pro-fibrotic M2, and various mixed-phenotype subsets, each with unique gene expression profiles and pathological functions, overturning the traditional static M1/M2 dichotomy (13, 50). Spatial transcriptomics further demonstrated that M1 pro-inflammatory macrophages are enriched in the core regions of inflammatory infiltration, whereas M2 pro-fibrotic macrophages aggregate in matrix-remodeling areas and overlap with fibrotic foci (111). Multi-omics integration and trajectory inference allow precise mapping of spatial cell-cell interactions and molecular networks, revealing the dynamic transition of macrophages from M1 to pro-fibrotic M2 phenotypes. Future studies should integrate single-cell transcriptomics, spatial transcriptomics, proteomics, and metabolomics to construct a panoramic atlas of the SS glandular microenvironment, providing a theoretical basis for the discovery of specific targets and biomarkers.

## 9 Conclusions and perspectives

In summary, the pathological progression of SS is essentially a result of spatiotemporal dysregulation of macrophage polarization. Early M1 polarization drives inflammation initiation; mid-stage M1/M2 interplay sustains chronic inflammation; and late-stage aberrant M2 polarization drives glandular fibrosis. TCM, with its multi-target holistic regulation, can effectively restore macrophage polarization balance and offers a new avenue for long-term intervention. However, several critical knowledge gaps remain: the origin, differentiation, and spatiotemporal distribution of macrophage subsets are not well defined; the regulatory effects of local matrix mechanical forces, metabolic microenvironment, and direct cell-cell contact signals on polarization require further investigation; the core drivers of macrophage phenotype transition during fibrosis are not yet identified; causal evidence for polarization imbalance in human samples is lacking; the material basis, core targets, and spatiotemporal regulatory mechanisms of TCM in modulating polarization are unclear; and gland-targeted delivery systems and polarization-specific biomarkers have not entered clinical development.

To address these issues, future basic research should integrate single-cell multi-omics, gene editing, and glandular organoid models to deeply analyze subset heterogeneity and microenvironmental crosstalk. TCM research should use multi-omics technologies to identify active components and their molecular pathways, and conduct large-scale RCTs to validate clinical efficacy and safety. Clinical translation should focus on developing targeted delivery systems and precision-modulating drugs, and promoting early intervention, spatiotemporally differentiated therapy, and integrated TCM-western medicine models. In the context of precision medicine, individualized stratification based on patients’ macrophage polarization subtypes, microenvironmental features, and degree of fibrosis should guide tailored intervention regimens. Through integration of multidisciplinary cutting-edge technologies, basic research findings on targeting macrophage polarization can be efficiently translated into safe, effective, and personalized therapeutic strategies for SS.
